# Alternation of the Rich-Club Organization of Individual Brain Metabolic Networks in Parkinson’s Disease

**DOI:** 10.3389/fnagi.2022.964874

**Published:** 2022-07-08

**Authors:** Liling Peng, Zhimin Zhang, Xiaofeng Chen, Xin Gao

**Affiliations:** ^1^Shanghai Universal Medical Imaging Diagnostic Center, Shanghai, China; ^2^Department of Blood Transfusion, Xiangya Hospital, Central South University, Changsha, China; ^3^College of Mathematics and Statistics, Chongqing Jiaotong University, Chongqing, China

**Keywords:** metabolic brain network, FDG-PET, rich club, JS divergence, Parkinson’s disease

## Abstract

**Objective:**

The diagnosis of Parkinson’s disease (PD) remains challenging. Although ^18^F-fluorodeoxyglucose (^18^F-FDG) positron emission tomography (PET) has revealed the metabolic abnormalities associated with PD at systemic levels, the underlying rich-club organization of the metabolic connectome in these patients remains largely unknown.

**Materials and Methods:**

The data of 49 PD patients and 49 well-matched healthy controls (HCs) were retrieved and assessed. An individual metabolic connectome based on the standard uptake value (SUV) was built using the Jensen-Shannon Divergence Similarity Estimation (JSSE) method to compare the rich-club properties between PD patients and HC.

**Results:**

Our results showed the rich-club organization of metabolic networks (normalized rich-club coefficients > 1) in the PD and HC group were within a range of thresholds. Further, patients with PD demonstrated lower strength and degree in rich-club connections compared with HCs (strength: HCs = 55.70 ± 8.52, PDs = 52.03 ± 10.49, *p* = 0.028; degree: HCs = 56.55 ± 8.60, PDs = 52.85 ± 10.62, *p* = 0.029), but difference between their feeder and local connections was not significant.

**Conclusion:**

Individual metabolic networks combined with rich club analysis indicated that PD patients had decreased rich club connections but similar feeder and local connections compared with HCs, indicating rich club connections as a promising marker for early diagnosis of PD.

## Introduction

Parkinson’s disease (PD) is the most commonly diagnosed chronic degenerative neurological dyskinesia. It affects more than 6 million individuals worldwide and significantly impacts the life quality of elder people by causing irreversible brain damage (Feigin et al., 2019; Li et al., 2021a). According to statistics, advancing age represents the most significant risk factor for developing PD. Genetics were also shown to play pivotal roles in PD pathogenesis, with over 90 independent risk variants identified (Blauwendraat et al., 2020). The pathological hallmark of PD is dopaminergic neuronal degeneration in the substantia nigra pars compacta with abnormal intracellular α-synuclein aggregation within the cytoplasm of neuronal cells in several different brain regions (Braak et al., 2002). The major motor symptoms of PD include akinesia, slowed movements, uncontrolled tremors, rigid muscles and postural instability, while non-motor symptoms include sleep disorders, dysosmia and cognitive disturbance (Tolosa et al., 2021). Despite important advancements that have been made in recent years in understanding the pathophysiology of PD, its underlying mechanisms of occurrence remain largely unknown, and early diagnosis remains challenging.

In recent years, significant focus has been placed on decoding the underlying neuroimaging mechanisms of PD via multimodal magnetic resonance imaging (MRI)(Mahlknecht et al., 2010). Functional neuroimaging techniques were used to localize abnormality in neuronal activities and explore the impact of the disease on brain networks combined with topological methods (Li et al., 2021b). The radiotracer ^18^F-fluorodeoxyglucose (^18^F-FDG), a radiotracer acting as a glucose analog, provided an index for cerebral glucose metabolism combined with positron emission tomography (PET). Previous literature on ^18^F-FDG PET evaluating brain metabolic differences between PD and normal cases identified decreased metabolism in the parieto-occipital and latero-frontal regions and increased metabolism in the sensorimotor regions, putamen, thalamus and cerebellum of PD patients (Peng et al., 2014). In a study that investigating the efficacy of FDG-PET imaging as a biomarker for determining PD progression, the investigators found that patterns of hypometabolism detected at baseline with FDG PET had an 85% sensitivity and 88% specificity in predicting the risk of progression to cognitive impairment (Pilotto et al., 2018). These analyzes were based on voxel levels to localize abnormal metabolic brain regions. However, brain regions with similar metabolism activities are considered functionally interconnected, forming a metabolic network. Using ^18^F-FDG PET imaging and graph theoretical analysis, Ko et al., 2018 assessed the topological properties of the metabolic networks in PD patients and an experimental non-human primate model. They found a metabolically active core with pathological exaggeration of small-worldness in the hypermetabolic regions (i.e., subcortical regions) of human and non-human PD models (Ko et al., 2018).

Recently, research in neuroimaging suggested rich-club organization as an important topological property existing in brain networks, whereby high-degree of dense interconnections were found between highly connected regions within the brain. These highly connected regions are called “hub” regions and the other brain regions are called “peripheral” regions. The edges of each individual’s brain network were classified as rich-club connections (hub-hub regions linking), local connections (peripheral-peripheral regions linking), and feeder connections (regional-peripheral regions linking). The structural and functional networks of rich-club provide a novel way to examine the potential underlying mechanisms of neurodegenerative diseases and neurodevelopmental disorders (Ray et al., 2014; Shu et al., 2018, Chen et al., 2020; Peng et al., 2022). Currently, no studies have investigated the alternations in rich-club organizations of the metabolic networks using ^18^F-FDG PET mapping in PD. This study used ^18^F-FDG PET data to construct an individual metabolic network and applied graph theory approaches to explore rich-club organization alterations in the metabolic connectome of patients with PD.

## Materials and Methods

### Participants

This study consisted of PD patients and healthy controls (HCs), enrolled between January 2018 and December 2019, and matched for similar age, education and gender to obtain normative data (Li et al., 2021a); resulting in 49 PD patients (33 male and 16 female participants; age, 53.94 ± 11.16 years) matched with 49 HCs. All HCs had no neurological impairment/disease or head injury. PD was diagnosed with idiopathic PD based on the International Parkinson and Movement Disorder Society (MDS) diagnostic criteria (Postuma et al., 2015). All PD cases were consecutively enrolled. Cases were excluded if: (1) had a history of head injury or stroke; (2) any psychological disorder; (3) underwent intracranial surgery, and; (4) had a past history of substance use disorder. All participants consented to this study. The study was performed following the Declaration of Helsinki and the protocol received approvement from the Ethics Committee of Xiangya Hospital, Central South University.

### ^18^F-FDG PET Imaging and Evaluation

^18^F-FDG PET imaging was performed using the Discovery Elite PET/computed tomography (CT) scanner (GE Healthcare) at the PET Center of Xiangya Hospital. The participants were advised to rest for 45–60 min with their eyes closed in the supine position on the PET scanner bed. The PET/CT scanning was conducted for 10 min using 3-dimensional (3D) mode after ^18^F FDG (3.7 MBq/kg) injection (intravenous). The images were reconstructed using the ordered subset expectation maximization algorithm with 6 iterations and 6 subsets method. Prior to image preprocessing, the DICOM images of PET were transformed into NIfTI images using the dcm2nii software (version 12^1^). Preprocessing of PET images was performed with the statistical parametric mapping (SPM) software^2^ implemented on MATLAB. Individual ^18^F-FDG PET image volumes were manually reset to the origin of 3-dimensional standard stereotactic Montreal Neurological Institute (MNI) spaces. The image intensity of all participants was globally normalized for a homogeneous comparison. A standard uptake value (SUV) image was generated for each participant. Then, the automated anatomical labeling (AAL) atlas was applied to parcellate all SUV images into 90 ROIs (45 per hemisphere without cerebellum), which were then defined as nodes of the individual metabolic network.

### Individual Jensen-Shannon Divergence Similarity Estimation (JSSE) Metabolic Network Construction

After obtaining the preprocessed SUV images, the individual metabolic network was constructed. Based on the current distribution-divergence-based method (Wang M. et al., 2020), the metabolic similarity was denoted as the edge between ROIs. In particular, the edges of networks were defined as the similarity between probability distributions of SUVs of all voxels of any ROI pairs. Using validated methodologies in previous literature (Li et al., 2021a; Tang et al., 2021, Li et al., 2022), the Jensen-Shannon (JS) divergence was used to perform similarity measurements using the following equation:


(1) $$D_{JS}\left(\mathbb{P}||\mathbb{Q}\right)=\frac{1}{2}\left[D_{KL}\left(\mathbb{P}||𝕄\right)+D_{KL}\left(\mathbb{Q}||𝕄\right)\right]$$

Here, ℙ and ℚ refer to probability density functions (PDFs) of voxel intensities in ROI pairs; 𝕄=0.5×(ℙ + ℚ) and *D*_*KL*_(⋅|⋅) represent the KL divergence. The probability distributions of SUVs were estimated using kernel density estimation (Duong, 2007). It should be noted that a smaller divergence score indicated more similar PDFs between two ROIs for this adjacency matrix to describe pairwise metabolic connectivities, whereby the metabolic connection strengths between regions *i* and *j* was referred to corresponding element*sJSs*(ℙ||ℚ) in the adjacency matrix.


(2) $$JSs\left(\mathbb{P}||\mathbb{Q}\right)=\text{exp}\left(-D_{JS}\left(\mathbb{P}||\mathbb{Q}\right)\right)$$

### Rich-Club Organization

**FIGURE 1:**
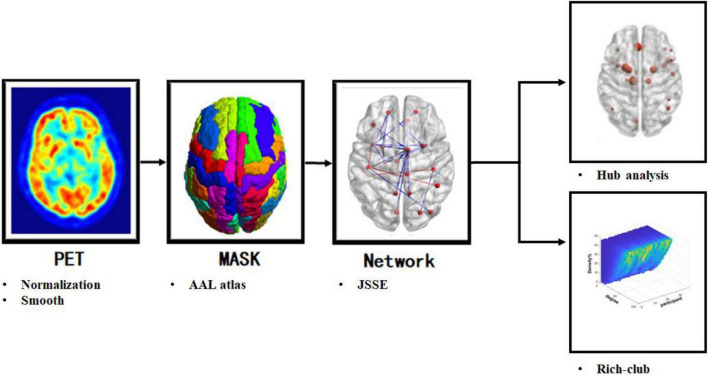
The flow diagram of positron emission tomography (PET) images preprocessing, individual metabolic network construction and analyzes. The flow diagram shows the preprocessing of PET images, construction of the individual metabolic network based on JSD, hub analysis and rich-club analysis.

Rich-club organizations, characterized by highly interconnected brain regions, represent an important network topology providing a structural frame to efficiently integrate and separate information processing. A flow diagram of the image preprocessing, individual metabolic network construction, and rich club analysis are shown in Figure 1.

Based on the average weighted metabolic network of all participants, the rich club coefficient (φ) was determined, which was then normalized (φ_*norm*_) relative to a set of 1000 random networks from the sparsity of 0.1 to 0.4 with steps of 0.01. φ_*norm*_ > 1 over a range of degrees indicated presence of rich-club organizations in the metabolic networks. The top 14 highest degree brain regions, which corresponded to the highest-ranking 15% of the 90 regions based on the group average metabolic network, were called hub regions. Upon categorizing the whole brain regions into hub and peripheral regions, the edges of each individual’s metabolic network were classified as rich-club connections (hub-hub regions linking), local connections (peripheral-peripheral regions linking), and feeder connections (regional-peripheral regions linking). The GRaph thEoreTical Network Analysis (GRETNA) toolbox^3^ was used to perform rich-club organization analysis.

### Statistical Analyzes

All analyzes were performed on the Statistical Package for Social Sciences software (SPSS, version 22). Comparisons between groups in age and education were determined using the two-sample t-tests, while comparisons between groups in sex were performed the chi-square (χ^2^) test. The connectivity strength of the rich club, feeder and local connections between the PDs and HCs were compared using the two-sample *t*-test. *P* values < 0.05 represented significant difference (*p* value was uncorrected).

## Results

### Baseline Characteristics

In total, 49 HCs were matched with 49 PDs and included in this study. The participants’ baseline characteristics are shown in Table 1. They were comparatively similar in terms of education (*p* = 0.163), gender (*p* = 0.577), and age (*p* = 0.832).

**TABLE 1:** Baseline characteristics in Parkinson’s disease (PD) patients and healthy controls (HC).

Variables	HC (*n* = 49)	PD (*n* = 49)	*P*
Education (years)	13.44 ± 3.15	12.37 ± 4.06	0.163
Sex (male/female)	30/19	33/16	0.577
Age (years)	52.12 ± 9.84	53.94 ± 11.16	0.832
UPDRS-III score	NA	23.2	NA
Disease duration (months)	NA	60.2	NA

*HC, healthy controls; PD, Parkinson’s disease; UPDRS, unified Parkinson’s disease rating scale.*

### Rich-Club Organizations Between Parkinson’s Disease (PD) and Healthy Control (HC)

**FIGURE 2:**
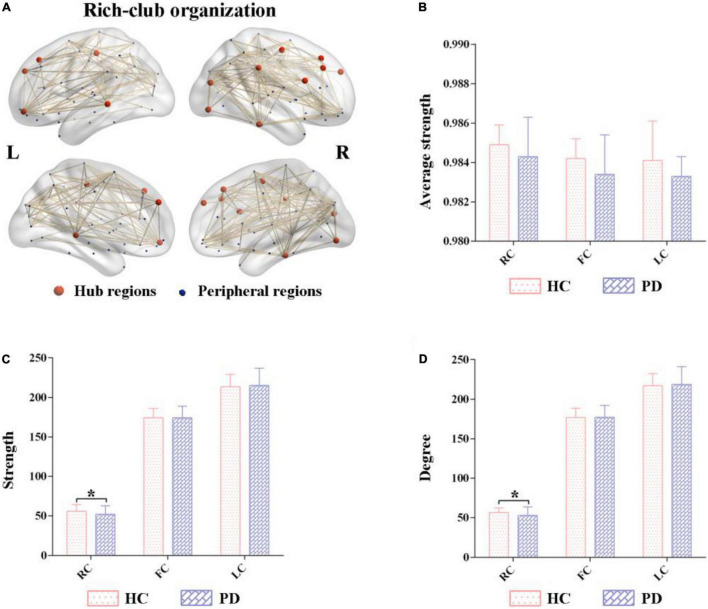
Altered rich-club organizations of metabolic networks in Parkinson’s disease (PD) and healthy control (HC). **(A)** The top 14 (15%) highest-degree nodes (hub regions) and the remaining 76 regions (peripheral regions). **(B–D)** Comparisons of the strength and degree of rich-club connections were significantly different between PD and HC. In contrast, their feeder and local connections were comparatively similar. PD, Parkinson’s disease; HC, healthy control.

A normalized rich-club coefficients greater than 1 was employed to assess the average group level of metabolic networks between HCs and PDs, and was found to range from 0.1–0.4 with steps of 0.01. The biggest normalized rich-club coefficient was found in the sparsity threshold of 0.11 (φ_*norm*_ = 1.108). Based on a network density at 11% in the average metabolic network, the hub nodes were identified by sorting the nodal degree. The top 14 (15%) highest-degree nodes were defined as hub regions and were primarily distributed in the prefrontal, lateral temporal and medial parietal regions, which was consistent with the findings from a previous study (Wang S. et al., 2020) (Figure 2A and Table 2). The remaining 76 brain regions were classified as peripheral regions (Figure 2A). Significant differences in the strength and degree of the rich club connections were found (Figures 2B–D). In detail, the PDs showed a decrease in strength and degree of rich club connections from HCs (strength: HCs = 55.70 ± 8.52, PDs = 52.03 ± 10.49, *p* = 0.028; degree: HCs = 56.55 ± 8.60, PDs = 52.85 ± 10.62, *p* = 0.029) (Figures 2C,D). In contrast, there were no significant differences in feeder and local connections between HCs and PDs (strength of feeder connections: HCs = 174.12 ± 11.91, PDs = 174.36 ± 14.50, *p* = 0.92; strength of local connections: HCs = 213.60 ± 15.40, PDs = 215.06 ± 21.89, *p* = 0.65; degree of feeder connections: HCs = 176.88 ± 11.55, PDs = 177.29 ± 14.70, *p* = 0.86; degree of local connections: HCs = 217.04 ± 15.32, PDs = 218.71 ± 22.27, *p* = 0.61; average strength of feeder connections: HCs = 0.984 ± 0.011, PDs = 0.983 ± 0.004, *p* = 0.62; average strength of local connections: HCs = 0.984 ± 0.011, PDs = 0.983 ± 0.004, *p* = 0.61) (Figures 2B–D).

**TABLE 2:** Hub regions of the metabolic network.

Labels	Regions	Anatomical classification
4	Superior frontal gyrus, dorsolateral	Prefontal
23	Superior frontal gyrus, medial	Prefontal
24	Superior frontal gyrus, medial	Prefontal
2	Precental gyrus	Frontal
9	Middle frontal gyrus, orbital part	Prefontal
90	Inferior temporal gyrus	Temporal
85	Middle temporal gyrus	Temporal
12	Inferior frontal gyrus, opercular part	Prefontal
52	Middle occipital gyrus	Occipital
64	Supramarginal gyrus	Parietal
3	Superior frontal gyrus, dorsolateral	Prefontal
8	Middle frontal gyrus	Prefontal
57	Postcentral gyrus	Parietal
54	Inferior occipital gyrus	Occipital

## Discussion

This study used 18F-FDG PET imaging data to construct individual metabolic networks based on the JSSE algorithm to investigate alterations in rich-club organizations of the brain metabolic connectome in PD patients. Our results showed lower strength and degree of rich club connections in patients with PD compared with HC, indicating that the hub regions of PD patients were more susceptible to metabolic stress and becoming functionally comprised; thus, providing an insight into the underlying mechanism of the metabolic connectome dysfunction in PD.

We are the first to reveal the rich-club organization of the metabolic network in patients with PD based on a constructed individual metabolic network using the novel JSSE method. JSSE was able to extend the asymmetrical KLSE to calculate a bounded and symmetrical divergence score of one probability distribution from another (Li et al., 2021a). The superiority of JSSE over KLSE includes two aspects. First, the JSSE ranges from 0 (identical) to 1 (maximally different), demonstrating a more accurate judgment in similarity when making comparisons between two groups, compared with KLSE, which ranges from 0 to +∞. Second, JSSE is symmetric, rendering easier visualizations of the connections between ROIs.

The brain network is composed of anatomically distinct regions indicative of integrated and segregated information processing to maintain the execution of neural functions. Network architectures showed the presence of a small high degree regions termed the “rich club,” which was “rich” in degree and formed a dense level of interconnections; constituting a pivotal role in the overall networking system (Van Den Heuvel and Sporns, 2011). Rich-club organizations are considered a keystone for global information integration in the brain (Van Den Heuvel et al., 2012). In this study, rich club regions were primarily distributed within the superior occipital, frontal, mid-frontal and inferior temporal gyrus, concording with previous literature (Wang S. et al., 2020). The normalized rich-club coefficient in the sparsity ranging from 0.1 to 0.4 with steps of 0.01 was higher than 1, indicating the existence of metabolic networks in the rich-club organization of PD patients. Our findings also showed that the rich club connections in PD patients had lower strength and degree compared with HC, while no significant difference in feeder and local connections between them was observed; suggesting disruptions in rich-club as an important step associated with PD progression. Previous studies have shown that rich-club components were highly vulnerable to damage, and due to their important roles in global communication, disturbances in these regions are more likely to manifest as symptoms in patients with PD (van den Heuvel and Sporns, 2013).

Our finding is consistent with results from a previous resting-state functional MRI study which also found disrupted nodal network measures in cortical hub regions in patients with PD compared with HC (Lin et al., 2018). Using diffusion tensor imaging to explore the neural mechanisms underpinning freezing of gait in patients with PD (Hall et al., 2018), Hall et al. found that PD freezers exhibited lower participation coefficients distributed in the subcortical, superior frontal and parietal regions than non-freezers. Additionally, several of these cortical regions were within the brain’s rich club region, indicating that freezing of gait in PD might be related to disturbances in the structural network topology between brain hub regions (Hall et al., 2018). These interesting findings suggest that PD primarily affects rich club connections at disease onset and could be an early biomarker for PD. The possible reasons for this observation could be: (1) higher connection strength between hub nodes could result in a higher probability of impairment (Hall et al., 2018), and (2) hub nodes are more susceptible to oxidative and metabolic stress (Fornito et al., 2015).

This study had some limitations worth discussing. First, the brain parcellation template that was used for constructing the brain metabolic networks may have, to a certain extent, affected the results of rich club analysis. Future studies should try implementing other brain schemes to confirm this study’s findings. Second, the study cohort was relatively small, and expanding the sample size using multicentre settings and longitudinal follow-up studies could yield higher-evidence level results. Lastly, considering that only ^18^F-FDG PET was used to assess metabolic networks in PD patients, a combination of multimodal imaging analysis could provide a more comprehensive understanding on the association between imaging biomarkers and disease symptoms in patients with PD.

## Conclusion

By using ^18^F-FDG PET imaging to construct individual metabolic networks combined with rich club analysis, this study found vulnerable rich-club connections in the metabolic network of PD patients. It could be considered as a promising imaging biomarker for the early detection of PD.

## Data Availability Statement

The original contributions presented in this study are included in the article/supplementary material, further inquiries can be directed to the corresponding author/s.

## Author Contributions

LP and ZZ designed the study and drafted the manuscript. ZZ collected the MRI data. XC and XG analyzed and interpreted the results of the data. XG revised the manuscript. All authors approved the final manuscript.

## Conflict of Interest

The authors declare that the research was conducted in the absence of any commercial or financial relationships that could be construed as a potential conflict of interest.

## Publisher’s Note

All claims expressed in this article are solely those of the authors and do not necessarily represent those of their affiliated organizations, or those of the publisher, the editors and the reviewers. Any product that may be evaluated in this article, or claim that may be made by its manufacturer, is not guaranteed or endorsed by the publisher.

## Footnotes

1
https://www.nitrc.org/projects/dcm2nii/

2
http://www.fil.ion.ucl.ac.uk/spm/soft-ware/spm12/

3
http://www.nitrc.org/projects/gretna/
